# Green Synthesis of Magnetic Nanoparticles of Iron Oxide Using Aqueous Extracts of Lemon Peel Waste and Its Application in Anti-Corrosive Coatings

**DOI:** 10.3390/ma15238328

**Published:** 2022-11-23

**Authors:** Nora Elizondo-Villarreal, Luz Verástegui-Domínguez, Raúl Rodríguez-Batista, Eleazar Gándara-Martínez, Aracelia Alcorta-García, Dora Martínez-Delgado, Edén Amaral Rodríguez-Castellanos, Francisco Vázquez-Rodríguez, Cristian Gómez-Rodríguez

**Affiliations:** 1 Universidad Autónoma de Nuevo León, Campus Cd. Universitaria, Ave. Universidad S/N, San Nicolás de los Garza 66455, Mexico; 2 Faculty of Engineering, University of Veracruz (Coatzacoalcos), Av. Universidad km 7.5 Col. Santa Isabel, Coatzacoalcos 96535, Mexico

**Keywords:** magnetite nanoparticles, green synthesis, lemon peel extracts, anticorrosive coatings, ecological method

## Abstract

Lately, the development of green chemistry methods with high efficiency for metal nanoparticle synthesis has become a primary focus among researchers. The main goal is to find an eco-friendly technique for the production of nanoparticles. Ferro- and ferrimagnetic materials such as magnetite (Fe_3_O_4_) exhibit superparamagnetic behavior at a nanometric scale. Magnetic nanoparticles have been gaining increasing interest in nanoscience and nanotechnology. This interest is attributed to their physicochemical properties, particle size, and low toxicity. The present work aims to synthesize magnetite nanoparticles in a single step using extracts of green lemon Citrus Aurantifolia residues. The results produced nanoparticles of smaller size using a method that is friendlier to health and the environment, is more profitable, and can be applied in anticorrosive coatings. The green synthesis was carried out by a co-precipitation method under variable temperature conditions. The X-ray Diffraction (XRD) and Transmission Electron Microscopy (TEM) characterization showed that magnetite nanoparticles were successfully obtained with a very narrow particle size distribution between 3 and 10 nm. A composite was produced with the nanoparticles and graphene to be used as a surface coating on steel. In addition, the coating’s anticorrosive behavior was evaluated through electrochemical techniques. The surface coating obtained showed good anticorrosive properties and resistance to abrasion.

## 1. Introduction

Magnetic Fe_3_O_4_ nanoparticles have gained much interest in nanoscience and nanotechnology. This interest is due to their unique physicochemical properties such as superparamagnetism [1], particle size, and morphology. Coupled with this, their low toxicity and the fact that they can be tolerated by the human body make them especially suitable for a wide range of applications in various areas such as engineering, biotechnology, environment, and medicine, among others, while increasing the demand for said nanoparticles. For example, laboratory studies have verified that Fe_3_O_4_ particles form a protective oxide layer that stops the advance of corrosive attacks on metallic structures [2,3]. They are often used as a contrast agent in nuclear magnetic resonance (NMR)-based imaging [4], proton exchange membranes [5], and sensors [6]. They are also used in the treatment of hyperthermia, acting as heat transmitters to concentrate the increase in temperature in a specific area without affecting healthy tissues and enhancing the activity of conventional therapies such as cancer therapy [7,8].

Many colloidal methods of synthesis have been approached to obtain metallic NPs, such as (i) homogeneous reduction in aqueous solutions [9] and (ii) phase transfer reactions with sodium citrate, hydrazine, NaBH_4_, and lithium triethyl borohydride (LiBEt_3_H) as reducing agents [10]. Both methods yield products with different physicochemical and structural characteristics [11]. Among these, the Polyol method has been reported to produce small NPs as the final product, with advantages such as easy changes in composition and surface modifiers. This technique does not require an additional reducing agent since the solvent by itself (ethylene glycol) reduces the metallic species [12]. In the literature, the one-step green co-precipitation synthesis of magnetite nanoparticles has been reported. The method uses naturally occurring biodegradable lemon-peel-based surfactants without any special reducing agent/capping agents. This green method uses water as a benign solvent and lemon peel extract as a surfactant/reducing agent. This green method reduces the temperature requirement, contrasting with the classical polyol method needing ethylene glycol and polyvinylpyrrolidone (PVP). The reactions with ethylene glycol are carried out in up to three hours and at temperatures up to 190 °C. PVP capping agent is removed from nanoparticles, using acetone to wash the particles three or more times [12]. Meanwhile, the green method uses only a solution of deionized water with ethanol. Furthermore, ethylene glycol and PVP are chemical compounds, while lemon peel is a natural waste and it is cheaper. In the green method, the NPs’ size and shape can be controlled by varying the concentration of lemon peel extracts and the temperature, and the reaction time is shorter. These natural components allow the synthesis of ultra-small magnetite NPs with very narrow distribution at relatively low temperatures.

Magnetite can be synthesized by various methods, including ultrasound irradiation [13], sol-gel [14], thermal decomposition [15], and co-precipitation [16]. Thermal decomposition and co-precipitation are the most commonly used. The thermal decomposition route relies on the pyrolysis of the organic precursors of iron, such as Fe(CO)_5_ [17] and Iron(III) acetylacetonate Fe(acac)_3_ [15,18]. However, Fe(CO)_5_ and Iron(III) acetylacetonate Fe(acac)_3_ are toxic and may limit the application of magnetite for medical applications.

Co-precipitations based on the hydrolysis of a mixture of Fe^2+^ and Fe^3+^ ions are used to fix the A-to-B molar ratio in the inverse spinel structure. In this method, Fe^2+^ and Fe^3+^ ions are generally precipitated in alkaline solutions, such as ammonium hydroxide, potassium hydroxide, or sodium hydroxide. Usually, the syntheses are performed at 70–80 °C [16] or higher temperatures [19].

The most common chemical method to obtain magnetic nanoparticles of Fe_3_O_4_ is the co-precipitation based on the hydrolysis of iron salts, which are generally precipitated in alkaline solutions in the presence of NH_4_OH, KOH, NaOH, or other chemicals toxic to the environment and human health [20,21,22,23,24]. For the maghemite nanoparticles to be obtained, it is necessary to make variations to the above method. In fact, a second oxidation to the magnetite nanoparticles is needed [22,23]. The process of modification or making a second synthesis to produce the maghemite is at the direct expense of materials and infrastructure.

There are a lot of different compounds present after extraction: plants contain a complex network of antioxidant metabolites and enzymes that work together to prevent oxidative damage to cellular components. Isolated quercetin [25] and polysaccharides have been used for metallic nanoparticle synthesis. Lemon peel extract contains chemically different compounds: polyphenols, flavonoids, sterols, triterpenes, triterpenoid saponins, beta-phenylethylamines, tetrahydroisoquinolines, reducing sugars such glucose and fructose, and proteins in all extracts [26,27,28,29].

Lemon peel extract contains natural compounds such as ascorbic acid, citric acid, minerals, flavonoids, and essential oil. The major compounds are limonene, terpinene, and neral, followed by *trans*-verbenol, decanal, and others [30,31].

The principal extract of lemon peel is D limonene (*Citrus volkameriana*) at approximately 78%. D limonene is a novel green surfactant that directly influences the nucleation and growth of nanoparticles. Therefore, lemon peel waste was used in this work. In addition, citrus fruit fiber also contains bioactive compounds, such as polyphenols, the most important being vitamin C (or ascorbic acid). This compound is an antioxidant used as a reducing agent in nanoparticle synthesis [32,33,34,35,36,37].

On the other hand, coatings can work as thermal barriers, changing the metal´s surface properties, such as the friction reduction between the surfaces that might be in direct contact with them. They also help prepare the surface or a part that has experienced a wear phenomenon. Furthermore, one of its most important functions is to serve as an anticorrosive barrier that may reduce the direct contact that the surface of the material can have with the environment and, in turn, provide new surface properties to it [38].

The use of magnetite nanoparticles can be diverse due to their versatility, easy handling, and stability against other nanoparticles and coupling matrices. In the present work, magnetite nanoparticles were combined to produce a composite material with anticorrosive characteristics. These nanoparticles had a barrier effect on the metal´s surface against the environment.

On the other hand, corrosion can be seen in the early, medium, and long-term life of metals. It depends on existing conditions impacting a lower- or higher-speed phenomenon. The corrosion phenomena are so diverse that almost no metal is exempt from them. This phenomenon ranges from an aesthetic material change, fracture initiation, loss of mechanical resistance in parts of machinery or structures, and leaks until fatal material destruction. The corrosion consequences are considerable economic losses, detrimental environmental effects, and in some cases, human diseases.

For corrosion to occur, besides oxygen, there must be a metal to be oxidized (anode), a generally conductive electrolytic solution (formed by salts and moisture from the environment), and a path leading to another metal or another part of the same material (cathode) that closes the circuit [39,40]. In this way, if one of these four elements is blocked or removed, corrosion will not occur.

Corrosion is a phenomenon that affects technology and also the economy. Therefore, it should be analyzed from these two perspectives. The direct corrosion cost due to industrial activities is estimated as playing a significant role in the Gross National Product, around 3% for most countries [41,42,43].

Some actions can be taken to protect metals from corrosion: connecting the metal to a sacrificial anode, applying galvanized or zinc-coated steel coatings, allowing a film (metal oxide) to form naturally on the material, and using corrosion inhibitors on the electrolytic medium to which they are exposed through organic or inorganic anticorrosive coatings, among other actions that achieve their goal. These actions aim to prolong the useful life of the material and prevent malfunctions due to corrosion. Choosing one of these will depend on economic factors and the benefits that the material offers.

There are several methods for evaluating the corrosion resistance of coatings. For quality control, the most used at an industrial scale is the salt spray test (ISO 9227: 2012). This test allows us to know the durability of the coating under an aggressive environment. However, some corrosion factors cannot be measured using this test.

Since the corrosion is mainly electrochemical in nature and the salt spray test has limitations, there are electrochemical techniques that provide other types of information that can be very useful, such as the corrosion rate. A clear advantage of these techniques is that corrosion resistance responses can be obtained in a shorter time than the salt spray test. For this purpose, the electrochemical tests of open circuit potential and potentiodynamic polarization curves will be carried out.

In this regard, the current work presents a green synthesis that allows us to obtain magnetite nanoparticles in a single step using extracts of lemons waste Citrus Aurontifolia Swingle [44]. Green synthesis is a revolutionary way to focus conventional chemical syntheses toward something more profitable and friendlier to health and the environment. Furthermore, plant extracts are particularly promising for green products as they are freely available, inexpensive, and offer easy use and scalability [45].

## 2. Materials and Methods

### 2.1. Green Synthesis of Magnetic Nanoparticles

The green synthesis of the magnetic nanoparticles (Fe_3_O_4_ and γ-Fe_2_O_3_) was carried out following the chemical co-precipitation method in an aqueous medium, using extracts of green lemon waste. A 0.01 M Fe ion solution was prepared by dissolving ferrous and ferric chlorides in deionized water. A 1:2 molar ratio of Fe^2+^/Fe^3+^ was used, the first one of iron(II) chloride tetrahydrate (FeCl_2_·4H_2_O) CAS: 13478-10-9 and the second one of iron(III) chloride hexahydrate (FeCl_3_·6H_2_O) CAS: 10025-77-1. The mixture was created in a three-neck flask with constant stirring for 20 min at different temperatures (see Table 1). Then, 20 mL of lemon extract at room temperature was added and left to be stirred for 20 more minutes. Finally, a sodium hydroxide (NaOH) CAS: 1310-73-2 solution of 0.02 M was added slowly until a pH of ~12 was reached. The reaction solution was mechanically stirred at 600 rpm. After the charging of the reactor with NaOH was completed, stirring continued for 2.5 h. After this reaction time, the solution was decanted, allowing the particles to be washed with deionized water. This procedure was repeated three times, and then the particles obtained were separated by a permanent magnet and dried overnight at 60 °C. The complete reaction that occurs can be seen in Equation (1).

2Fe^3+^ + Fe^2+^ + 8OH^−^ → Fe_3_O_4_ + 4H_2_O (1)

### 2.2. Lemon Waste Extract Preparation

**Figure 1:**
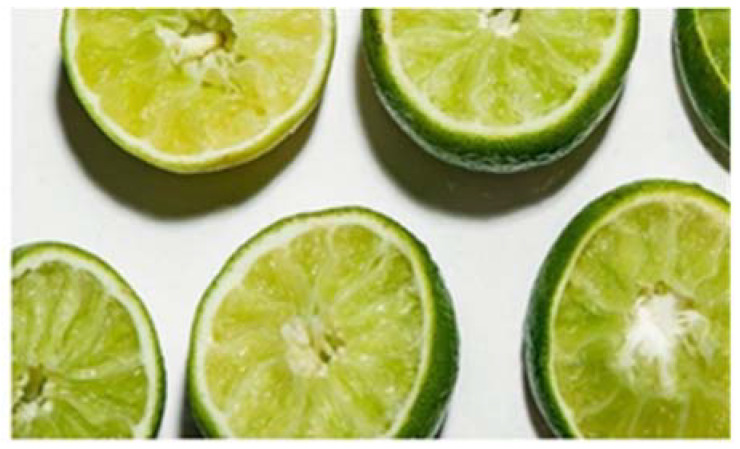
The lemon peel is a waste used to prepare the lemon extract.

For lemon extract preparation, the lemon peel waste was taken as shown in Figure 1, washed very well with deionized water CAS: 7732-18-5, and cut into small pieces.

Then, they were placed in a beaker with deionized water and left on the stirring plate for 25 min at a pH of ~3. The literature usually indicates that at pH 2–4, the reduction of Fe^3+^ ions is promoted [46]. Finally, the solution was filtered to remove the large pieces of lemon and then centrifuged several times to remove small impurities. The experimental parameters are shown in Table 1.

### 2.3. Compound Preparation

The Fe_3_O_4_–GO-Acqua100 compound was developed and used as an anticorrosive coating on the surface of ASTM A-662 steel samples. The materials used are detailed in Table 2.

Acqua 100: Acqua 100 Comex/fast-drying water-based anticorrosive enamel, lead-free. The color that was used was microphone 312-03.

ASTM A662: Standard Specification for Pressure Vessel Plates for Low and Moderate Temperature Service, prepared by the American Society for Testing and Materials or ASTM International.

The specifications of the reagents used are the following: GO (796034-1G/Sigma-Aldrich), DMF (CAS: 68-12-2/Sigma-Aldrich), Acqua 100 (Acqua 100-microphone 312-03/Comex).

A stoichiometry of 1:5 was used for the Fe_3_O_4_–GO ratio (GO is graphene oxide). The corresponding amount of GO CAS: 947-768-1 is placed in a container, and Dimethylformamide (DMF) was added to it, and it was then placed in an ultrasound bath for 30 min. The functionalized nanoparticles were added, and then the ultrasound process was carried out. After that, the solution was removed from the ultrasound and placed on a magnetic plate. It was kept for 5 h under constant agitation at 105 °C. Then, it was removed from the plate, filtered, and washed with ethyl alcohol CAS: Number: 64-17-5. Finally, it was dried in an oven at 60 °C for 24 h.

Acqua 100 acrylic enamel was used as a polymeric matrix to form the matrix/nanoparticle composite. For the suspension preparation, 3 bottles were used and then filled with Acqua 100, varying the number of particles added to the matrix, thus forming the combinations: R1 (coating without nanoparticles), R2 (coating with 0.5 wt. % of nanoparticles), and R3 (coating with 1 wt. % of nanoparticles) [47].

The addition of the nanoparticles into the matrix corresponds to the percentage of paint in each bottle (0%, 0.5 wt. %, and 1 wt. %). Then, the systems were subjected to mechanical agitation and ultrasound bath for 2 h to promote homogeneous dispersion throughout the matrix [48].

## 3. Results and Discussion

### 3.1. X-ray Diffraction

**Figure 2:**
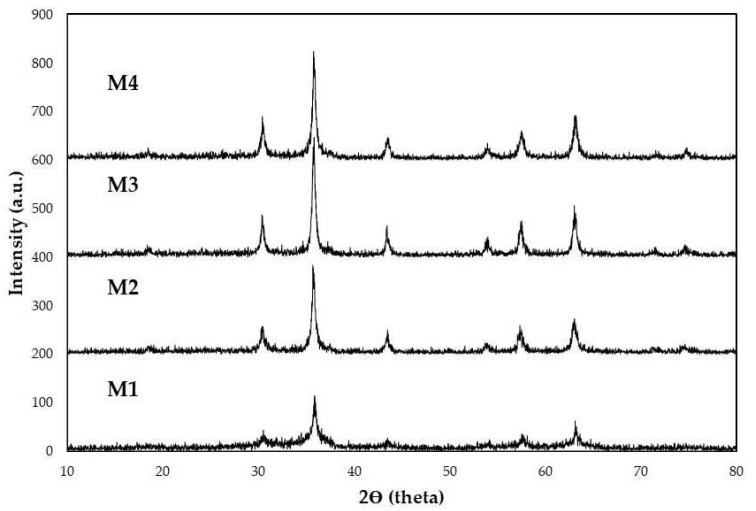
The diffractograms from samples M1, M2, M3, and M4 match the diffraction patterns of magnetite (Fe_3_O_4_).

Powder X-ray diffraction of chemically synthesized Fe_3_O_4_ nanoparticles is shown in Figure 2. Structural analysis of Fe_3_O_4_ was carried out using a PW3050/60 XPERT-3 diffractometer using PANalytical equipped with a Cu Kα source, a Bragg–Brentano geometry on one floor, and with 2θ steps of 0.01° in the range of 2θ from 20° to 80°.

### 3.2. Crystallite Size and Dislocation Density Å

The Scherrer formula was used to calculate the crystallite size “D”.

$$D=\frac{K\lambda }{\beta Cos\;\left(\theta \right)}$$

where *K* is the shape factor usually taken from 0.89 to 0.9, *λ* is the wavelength of the X-rays (source), which in our case is 1.54 Å, θ is the Bragg angle/half of diffraction angle, and *β* is the full width at the half maximum of reflexes, calculated in radians [49]. Some specific characteristics of samples of iron oxide are shown in Table 3.

The diffractograms obtained from each sample are shown in Figure 2.

### 3.3. D-Spacing Calculating

**Figure 3:**
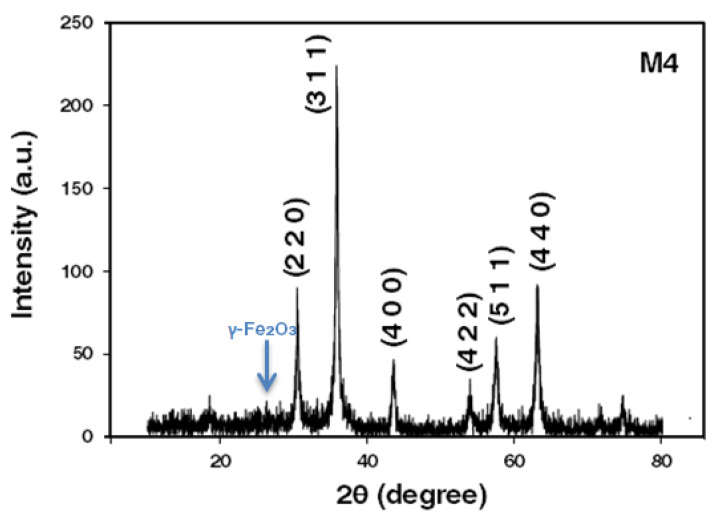
Diffractogram of magnetite nanoparticles obtained by the chemical co-precipitation and green chemistry method at 95 °C.

Samples of powdered magnetite nanoparticles were analyzed. Figure 3 shows the XRD magnetite of the M4 sample. Reflections are consistent with those reported in the JCPDS standard Magnetite No. 00-19-0629 [46,50]. The observed intensity index associated with each reflection is reported in Table 1. On the other hand, by calculating the lattice constant (a_0_), it turns to (311) and (400). The result of this calculation was a_0_ = 8.42 Å, which is among those reported for magnetite and maghemite (a_0_ = 8.351 and a_0_ = 8.399 Å). This fact may indicate that nanoparticles synthesized for this study show a crystal structure attributable to stoichiometric magnetite [51,52]. Two secondary peaks with low intensity were observed at 23.7 and 26.3 (2θ degree angle, using copper radiation). These peaks correspond to the characteristic maghemite species. Therefore, the dark brown powder obtained under the established conditions for this study showed the presence of maghemite, which corroborates the observed X-ray diffraction data [53].

Table 4 shows lattice spacing/distance between the atoms d_hkl_, the value of d is calculated using Bragg’s law.

2dsinθ=nλ

$$d=\frac{\lambda }{2sin\theta }\;\left(n=1\right)$$

Wavelength *λ* = 1.54 $\dot{A}$ for Cu K_α_.

### 3.4. SEM Microscopy

**Figure 4:**
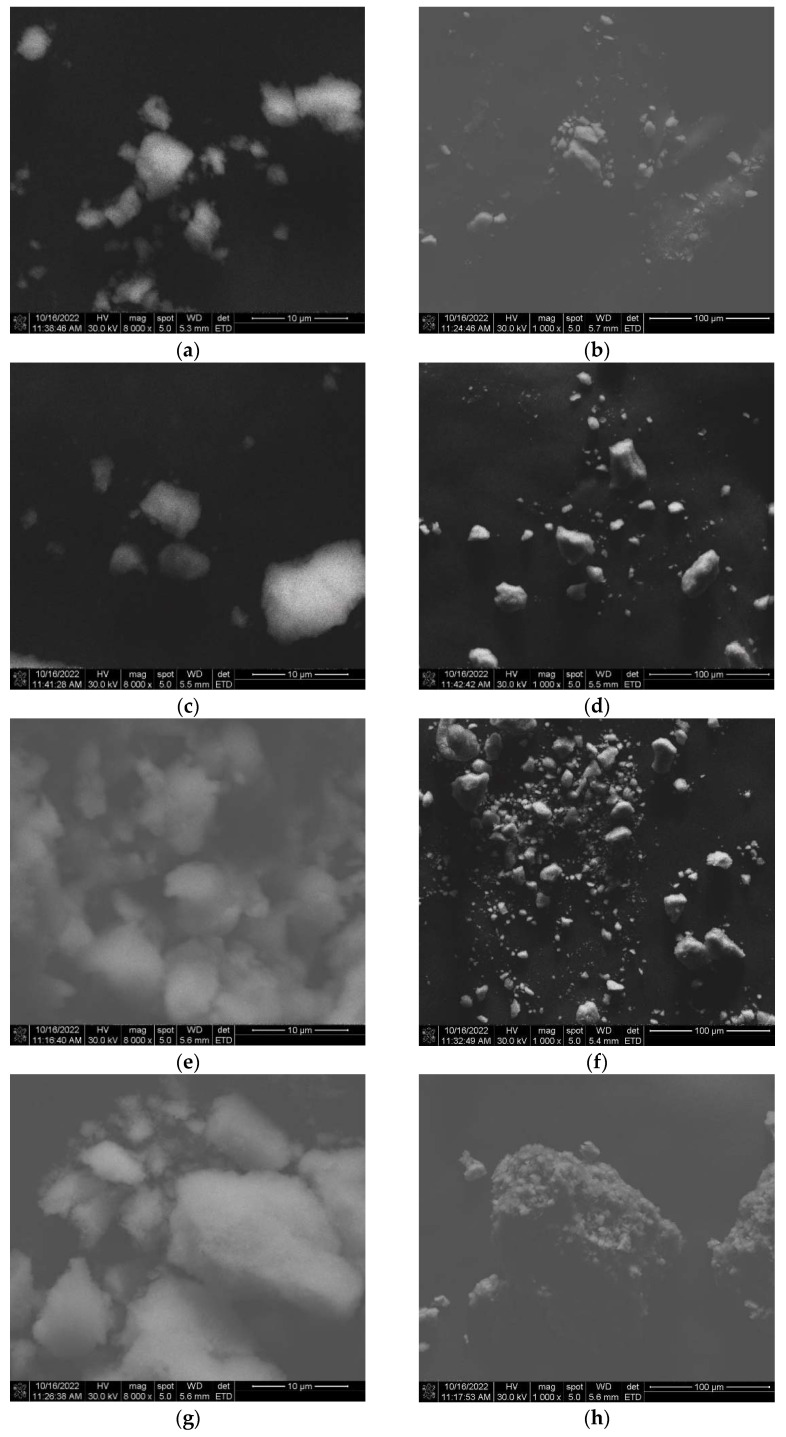
The SEM images of synthesized magnetite nanoparticles at magnifications of 10 µm and 100 µm for the samples M1 (**a**,**b**); M2 (**c**,**d**); M3 (**e**,**f**), and M4 (**g**,**h**).

Figure 4 shows SEM images of magnetite nanoparticles synthesized by the green co-precipitation method. Two magnifications were used to analyze the experimental samples (M1 to M4 samples). As observed, the reaction temperature influences the agglomeration phenomenon of the nanoparticles. Additionally, a certain uniformity and crystallinity were observed in the microstructure. The microstructural analysis can corroborate that this method can be effective for coating applications where many nanoparticles are used.

According to the literature, grain size growth (see Figure 4) is attributed to two mechanisms: Ostwald ripening (for more soluble materials) and oriented attachment (for less soluble crystals). In the former case, coarse particle formation is because of the dissolution of the smaller ones during the second due to merging of the smaller ones. Ostwald ripening occurs because coarse particles are more energetically steady than smaller particles. As is well known, temperature influences Ostwald ripening because of its set aside on the interfacial energy, development rate coefficients, and solubility. Meanwhile, oriented attachment occurs because the aggregation diminishes the interphase boundary and the whole surface of the system [54].

Temperature is one of the primary elements that contribute substantially to fixing the nanoparticles´ size, structure, and phase. The temperature is a parameter that influences the nanoparticles´ size, as a higher temperature favors a coarse particle size [55].

**Figure 5:**
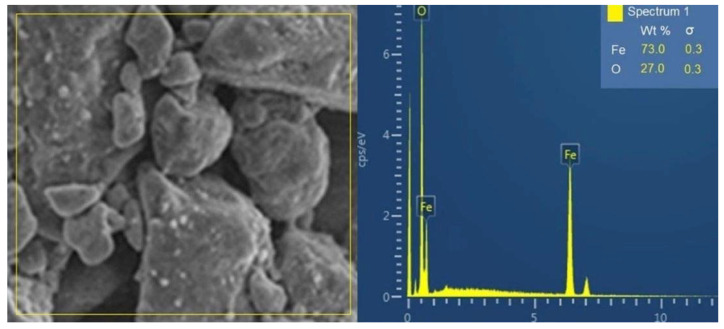
EDS spectra of the magnetite, where the presence of the oxygen and iron elements that constitute the sample were determined in percentages.

Figure 5 shows the EDS magnetite spectra, where the presence of the oxygen and iron elements that constitute the sample is indicated. The percentages of oxygen and iron are 27 wt. % and 73 wt. %, respectively, near the magnetite compound percentage (Fe_3_O_4_) with Fe = 72.35 wt. % and O = 27.64 wt. %. On the other hand, the chemical composition of the maghemite is Fe = 69.24 wt. % and O =30.06 wt. %. Therefore, this analysis allows one to have an idea of the iron phase present in the highest addition (M4 sample).

### 3.5. TEM Microscopy

**Figure 6:**
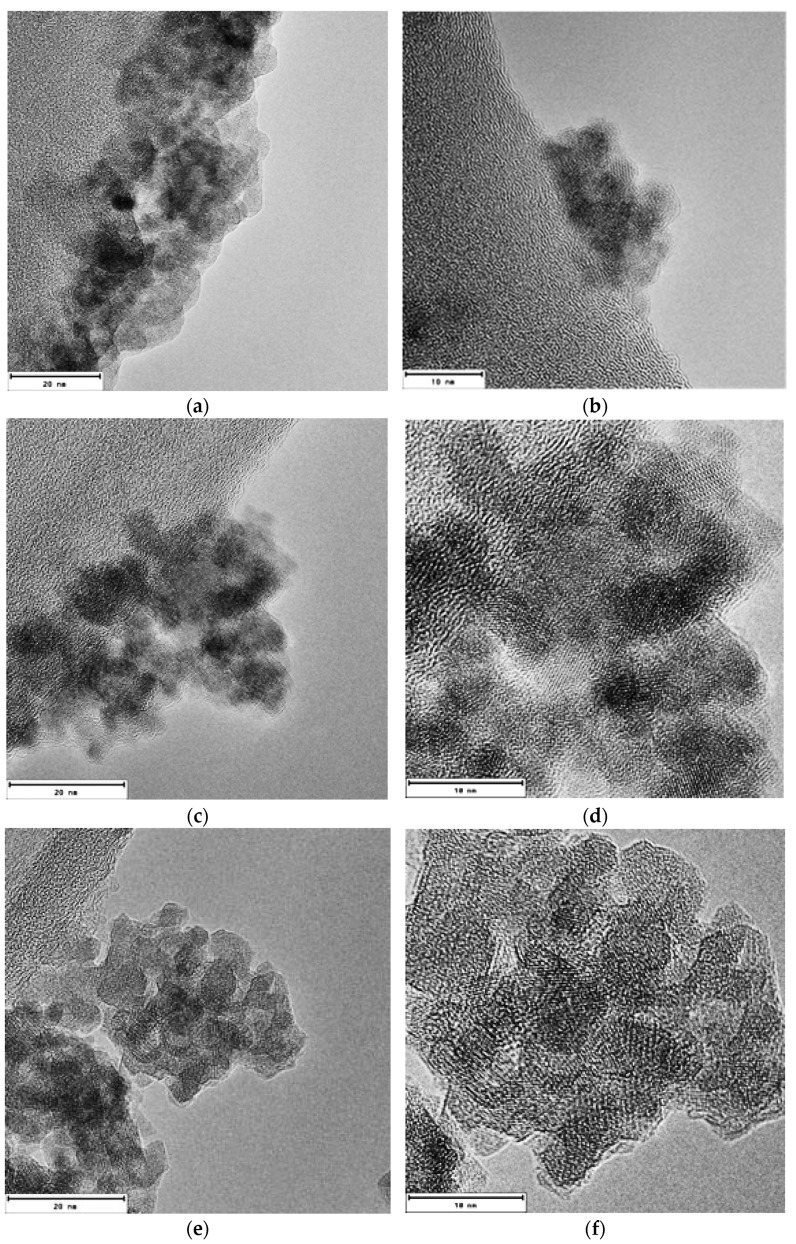
High-Resolution Transmission Electron Microscopy (HRTEM) images of the three representative samples M1 (**a**,**b**) M2 (**c**,**d**), and M3 (**e**,**f**), operating under magnifications of 20 nm and 10 nm; synthesized at a temperature of 25 °C, 55 °C, and 85 °C, respectively, by the green chemistry method.

The size and shape of the particles were studied by TEM microscopy using a 200 keV Josel-2000 FXII microscope. TEM samples were prepared by placing a drop of a magnetite nanoparticle suspension ethanol pure absolute for analysis ACS Supelco on a coated carbon grid, allowing the solvent to evaporate slowly at room temperature. The three representative samples synthesized by this method are shown in Figure 6. This image shows that the samples consist of nanoparticles with particle sizes smaller than 20 nm. Additionally, particle agglomerations are observed due to their superparamagnetic nature. The initial variation in temperature conditions extensively affects the morphology of the nanoparticles. At temperatures between 25 °C and 55 °C, nanoparticles with orthorhombic shapes were found. Meanwhile, at 85 °C and 95 °C, nanoparticles with rhombohedral morphologies were found.

In Figure 6, it can be seen that the magnetite nanoparticles tend to agglomerate. The presence of particles with a size distribution between approximately 7 and 12 nm, which are quasi-rounded and highly crystalline nanoparticles, can be appreciated in all these samples. From their hexagonal morphology observed in Figure 6b,d,f, it can be confirmed that magnetite nanoparticles were successfully synthesized.

**Figure 7:**
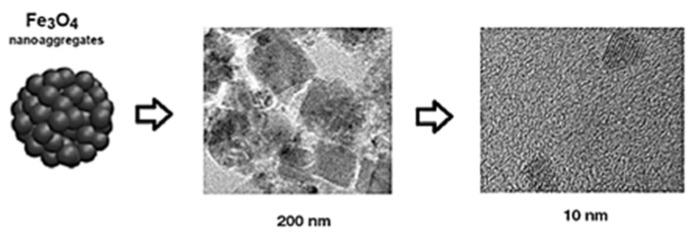
Scheme of nanoaggregates of Fe_3_O_4_ nanoparticles.

Figure 7 shows the scheme of nano-aggregates of Fe_3_O_4_ nanoparticles from 200 nm to 10 nm. These aggregates are a product of the magnetization of the nanoparticles.

**Figure 8:**
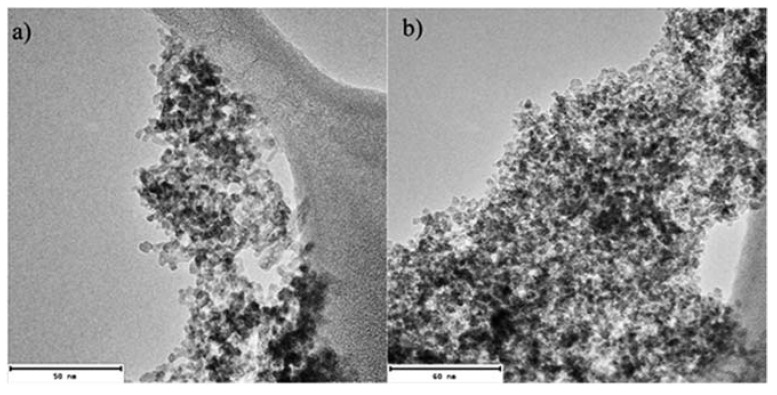
Images of HRTEM of obtained magnetite nanoparticles at a magnification of (**a**) 50 nm and (**b**) 60 nm of the sample. A uniform size distribution is observed in both images.

Figure 8 shows HRTEM images, on 50 nm and 60 nm scales, of the magnetite nanoparticles, where a very narrow and uniform size distribution is observed. According to the Scherrer equation (obtained from XRD characterization), about 3 nm particles with rhombohedral morphology are presented.

**Figure 9:**
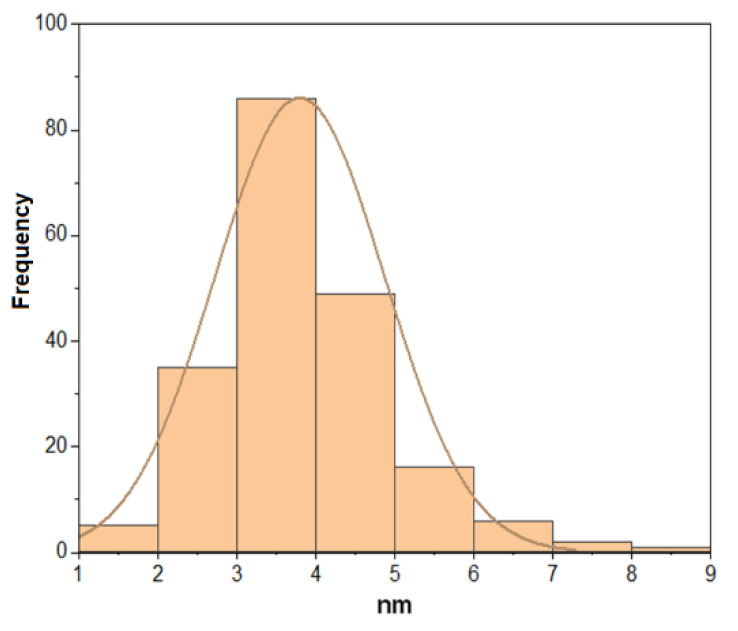
The particle size distribution plot at the nanoscale shows a size distribution of M1 sample. The histogram shows the frequency distribution of the nanoparticles with a normal distribution curve.

In the HREM images, magnetite particles at a nanometer scale are observed with a size less than 10 nm, so a graph of the diameter distribution of the nanoparticles was made with Origin and using the software ImageJ to realize the measurement of the nanoparticles (see Figure 9) [56,57]. Figure 9 shows a particle size distribution that fits a normal-type curve (orange line) with a mean diameter of 3.79 nanometers and a standard deviation of 1.07. This information matches the Scherrer equation data obtained from the M1 sample.

Characterization results by X-Ray Diffraction (XRD) and Transmission Electron Microscopy (TEM) showed that magnetite nanoparticles were successfully obtained. Orthorhombic and rhombohedral crystallinity and a narrow size distribution between 3 nm and 12 nm were observed. Meanwhile, literature has reported a size between 9 and 10 with NaOH utilization (without lemon peel) using the co-precipitation method [19].

As observed in the literature, the particle sizes obtained without using the extract (under the same synthesis conditions) are between 9 nm and 10 nm [19].

The influence of adding lemon peel extract is related to reaching a smaller particle size (~3 nm) than can be obtained using the conventional method with only NaOH.

As was observed, it is possible to replace some chemical compounds used in nanoparticle synthesis with some compounds from the lemon peel. These compounds act as surfactants, stabilizers, and reducers, forming the smallest nanometric particles. Specifically, D Limonene acts as a new and original surfactant agent [35], controlling the nucleation and growth process of nanoparticles. Thus, it obtained smaller particles than the conventional co-precipitation method without lemon peel extract.

**Figure 10:**
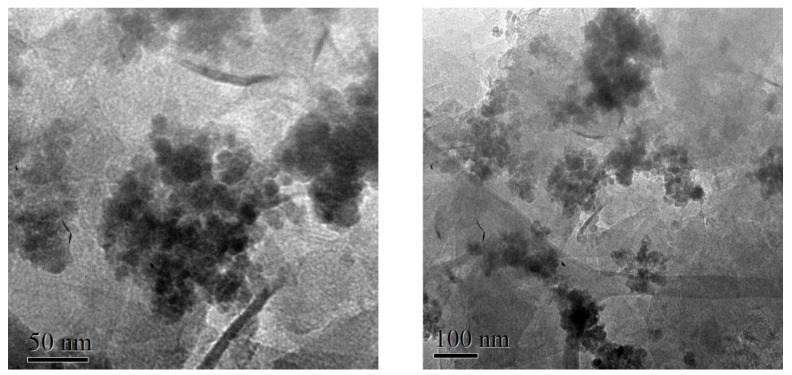
Images of TEM of obtained magnetite-graphene oxide composite, at 50 nm and 100 nm for the M1 sample.

Figure 10 shows the presence of magnetite nanoparticles deposited on graphene oxide sheets. A homogeneous magnetite distribution within the graphene oxide is also observed.

### 3.6. Magnetic Characterization

Superparamagnetism is a typical magnetism form of ferromagnetic or ferrimagnetic nanoparticles. In sufficiently small particles (of dimensions comparable to those of a magnetic domain of the corresponding massive material), the magnetic moments of the individual atoms are aligned. In this condition, the magnetization can change randomly due to the effect of temperature. This phenomenon is found in materials where the energy required for magnetic moment reversal of the particles is comparable to the lattice thermal energy. The typical time between two magnetic moment reversals is called the Néel relaxation time. Suppose an absence of external magnetic field condition. Then, the magnetization time of the nanoparticles would be much greater than the Néel relaxation time. Therefore, their magnetization would be zero, and the nanoparticles are said to be superparamagnetic. In this state, an external magnetic field can magnetize the nanoparticles, similar to a paramagnetic material. However, its magnetic susceptibility is much higher than that of paramagnetic materials.

The nanoparticles' magnetic properties were measured in a vibrant sample magnetometer. The conditions comprised a magnetic field strength of 9 kOe at room temperature.

**Figure 11:**
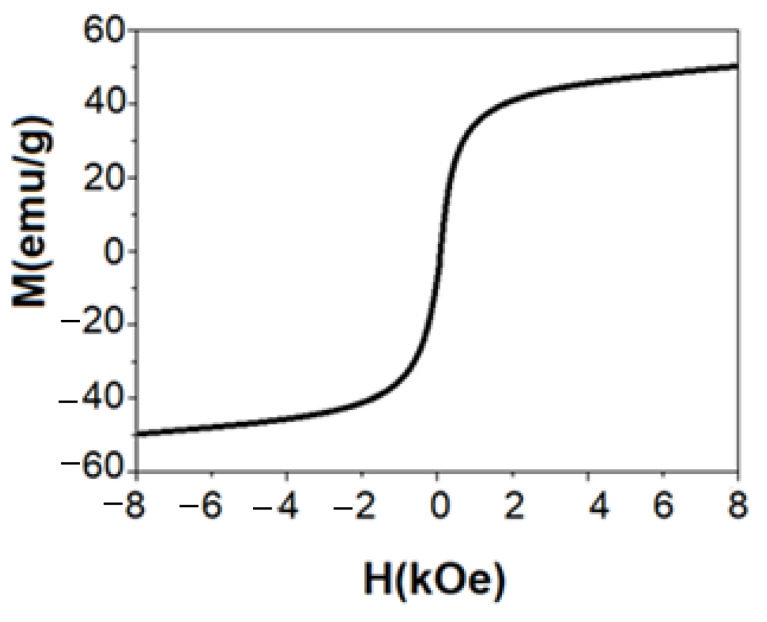
The magnetic curve of synthesized nanoparticles at room temperature.

Figure 11 shows the cycle of hysteresis of the magnetite nanoparticles. It is observed that the material has a typical superparamagnetic behavior. That is, it has zero coercivity. The saturation magnetization for the magnetite was 50 emu/g. The result shows that the magnetization value (Ms) is smaller than the bulk magnetite-Fe_3_O_4_ (93 emu/g). Three main reasons have been proposed to explain lower Ms of nanoparticles than that of their bulk state: (i) the presence of a non-magnetic layer on the NP´s surface (a dead magnetic layer), (ii) cations’ distribution, and (iii) surface spin disorder. The oxygen presence can lead to information about the other phases (e.g., maghemite) besides magnetite. However, the magnetite and maghemite phases have similar physical and structural properties. These conditions make it difficult to distinguish by the XRD analysis. Note that the magnetite phase is stronger than the maghemite phase in terms of magnetic properties, according to the literature. Furthermore, the transformation from bulk to nanostructure state (which is accompanied by the increase in surface/volume ratio and decrease in Ms) occurs by the intrinsic factor. Meanwhile, the oxidation factor depends on the synthesis conditions (e.g., the atmosphere type), which are relatively controllable [19,58,59,60,61,62,63,64].

**Figure 12:**
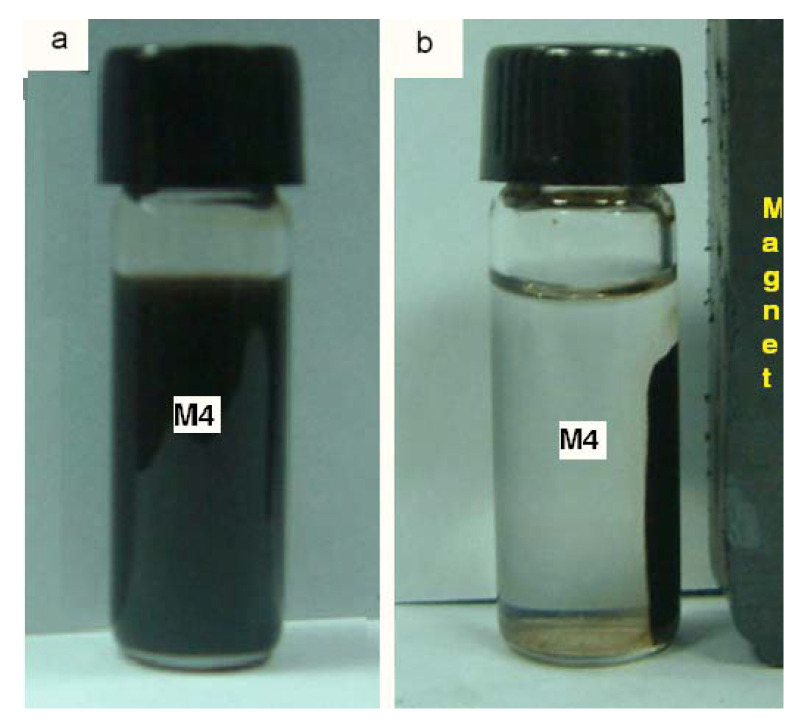
Image of magnetite nanoparticles: (**a**) suspension of Fe_3_O_4_, (**b**) by the effect of the magnet.

Figure 12 shows the M4 sample in solution and the M4 sample in the presence of a magnet. As the magnet approaches the sample, the magnetic domains align in the same direction toward the magnetic field generated by the magnet, so the particles move toward the direction of the magnet.

### 3.7. Obtaining and Applying the Coating

**Figure 13:**
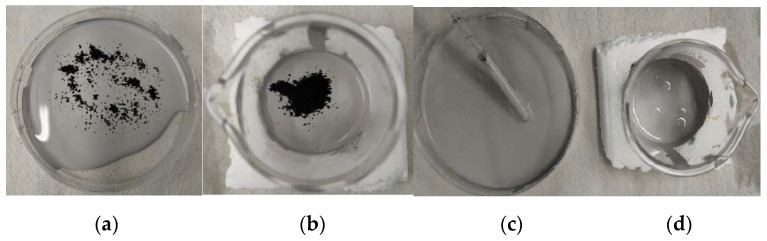
Incorporation of the nanoparticles into the Acqua 100 matrix. Obtaining the coating. From left to right the 0.5 wt. % and 1 wt. % coatings (**a**,**b**) before mixing and (**c**,**d**) after mixing.

Three experimental coatings were prepared: Acqua 100, Acqua 100 + 0.5 wt. % of NPs (GO-Fe_3_O_4_) and the last with 1 wt. % of NPs (GO-Fe_3_O_4_). Figure 13 shows (from left to right) the 0.5 wt. % and 1 wt. % coatings (a) and (b) before mixing, and (c) and (d) after mixing.

The obtained coating was applied to three groups of samples. Those coated with Acqua 100, Acqua 100 + 0.5 wt.% of NPs, and Acqua 100 + 1 wt.% of NPs. The method used was to immerse the piece in the coating for approximately 30 s and then let it dry for 48 h in a stable place at room temperature and 80% relative humidity.

**Figure 14:**
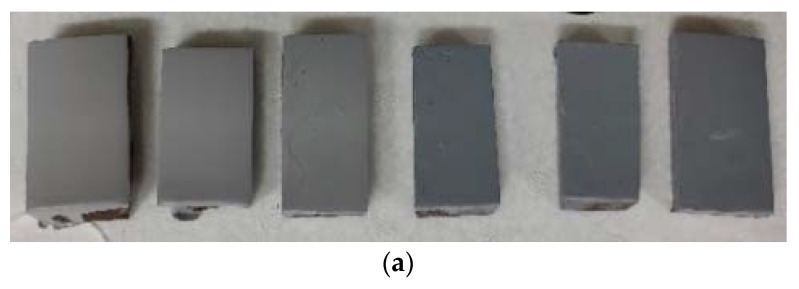

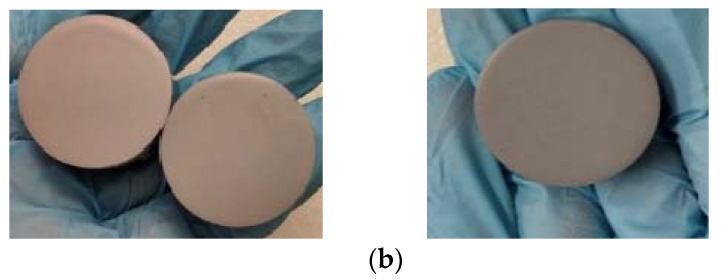
(**a**) Coated samples for tribology assays and (**b**) coated samples for electrochemical tests.

Figure 14a,b are representative coated samples obtained for the different tests subjected to electrochemical and tribology conditions. As the percentage of nanoparticles in the coating increased, its coloration became darker. This characteristic is typical of the nanoparticles used.

### 3.8. Electrochemical Tests

Metal corrosion processes in an aqueous medium allow the electrical quantities of potential (E), current (I), and resistance (R) to be related to the chemical and mass transport processes that take place during metallic corrosion [65]. As is well known, there are anodic and cathodic reactions during a corrosion phenomenon. These reactions take place simultaneously in different parts of the metal surface. Most of these reactions are not reversible since they do not correspond to equilibrium processes and involve some reactive species. Anodic reactions are usually metal dissolution or oxidation reactions. Meanwhile, cathodic ones are present in different reduction reactions depending on the nature and concentration of the species present in the medium.

The anodic potential is defined as the metal–metal ion ratio, while the cathodic potential is the type of reaction(s) that predominate(s) in the cathodic process. From the principle that the entire surface is equipotential, the metal undergoes corrosion and tends to present a single electrode potential. This condition provokes all the anodic and cathodic areas to polarize each other to reach a single value of potential. This is known as open circuit potential (OCP) or corrosion potential (Ecorr).

Polarization curves are used to determine the corrosion rate of a system from its relationship between current and potential. This technique consists of varying the potential (over potential) at a constant speed on the working electrode.

For the OCP tests carried out in the present work, the potential of the samples was allowed to stabilize for about 10 min to obtain a more stable thermodynamic potential reading. Once the potential stabilized, it was plotted as a function of time (for 15 min). The data of the metal equivalent weight (EW) and density (ρ) were also plotted.

The electrochemical tests of open circuit potential and potentiodynamic polarization curves were carried out according to the ASTM standards (G1, G5, G59, and G102), in addition to the sample preparation, assembly of the electrochemical cell, and determination of the corrosion values.

The assembly of an electrochemical cell with three electrodes was carried out where the following components were present:

- Auxiliary electrode: the platinum electrode was used.
- Reference electrode: the Saturated Calomel electrode (SCE) was used.
- Working electrode: the steel samples were used.

The tests were carried out with the Galvanostat/Potentiostat equipment, which is operated by automated software with two modules (CorrWare 2 and ZView 2). It is necessary to enter the conditions of the assembled cell into the program:

- Working electrode exposure area (cm^2^).
- Alloy equivalent weight: 27.92.
- Alloy density (g/cm^3^): 7.8.
- Stern-Geary constant (26 mV).
- Reference electrode.
- Initial potential.
- Final potential.
- Scan speed.

Before performing both tests, it is necessary to immerse them in an electrolyte solution of 3.5 wt. % of NaCl in water. The samples were exposed for 140 h to stabilize the conditions required for the tests.

**Figure 15:**
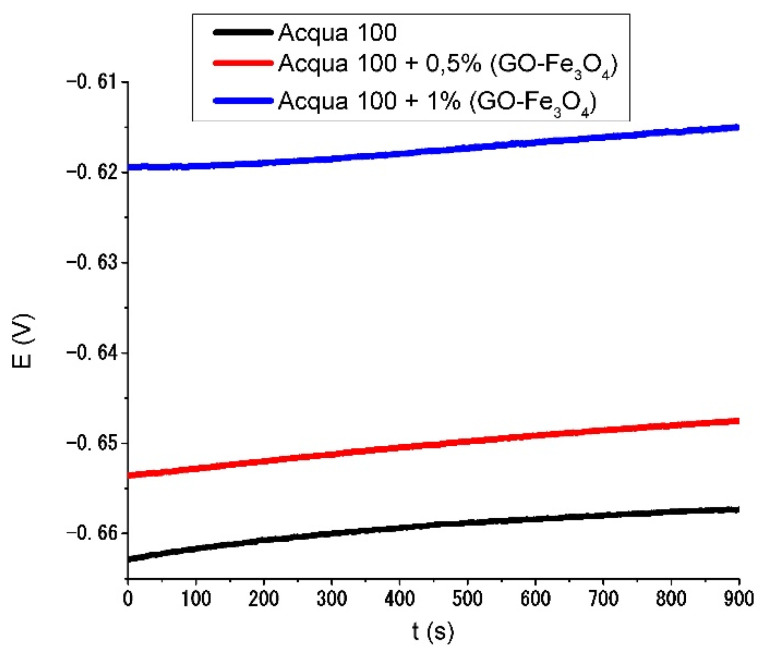
Evolution of the open circuit potential for the samples evaluated.

Figure 15 shows the results obtained from the open circuit potential test from the average value of the data obtained for each group tested: (Acqua 100, Acqua 100 + 0.5 wt. %, Acqua 100 + 1wt. %).

As observed in Figure 15, the samples that contained nanoparticles in their coating presented less negative potential values, which shows an improvement in the anticorrosive behavior of our formulation. The samples that contained 0.5 wt. % of GO-Fe_3_O_4_ nanoparticles in their coating registered higher average potential values (1.44%) than samples that did not contain nanoparticles in their coating. Meanwhile, the samples that contained 1 wt. % of GO-Fe_3_O_4_ nanoparticles showed an improvement in the corrosion’s average potential value (6.38%).

Table 5 shows the average values obtained from the samples tested for each group analyzed. The least negative value corresponds to the Acqua 100 + 1 wt. % NPs samples. For this type of test, this coating formulation reached the best results.

**Figure 16:**
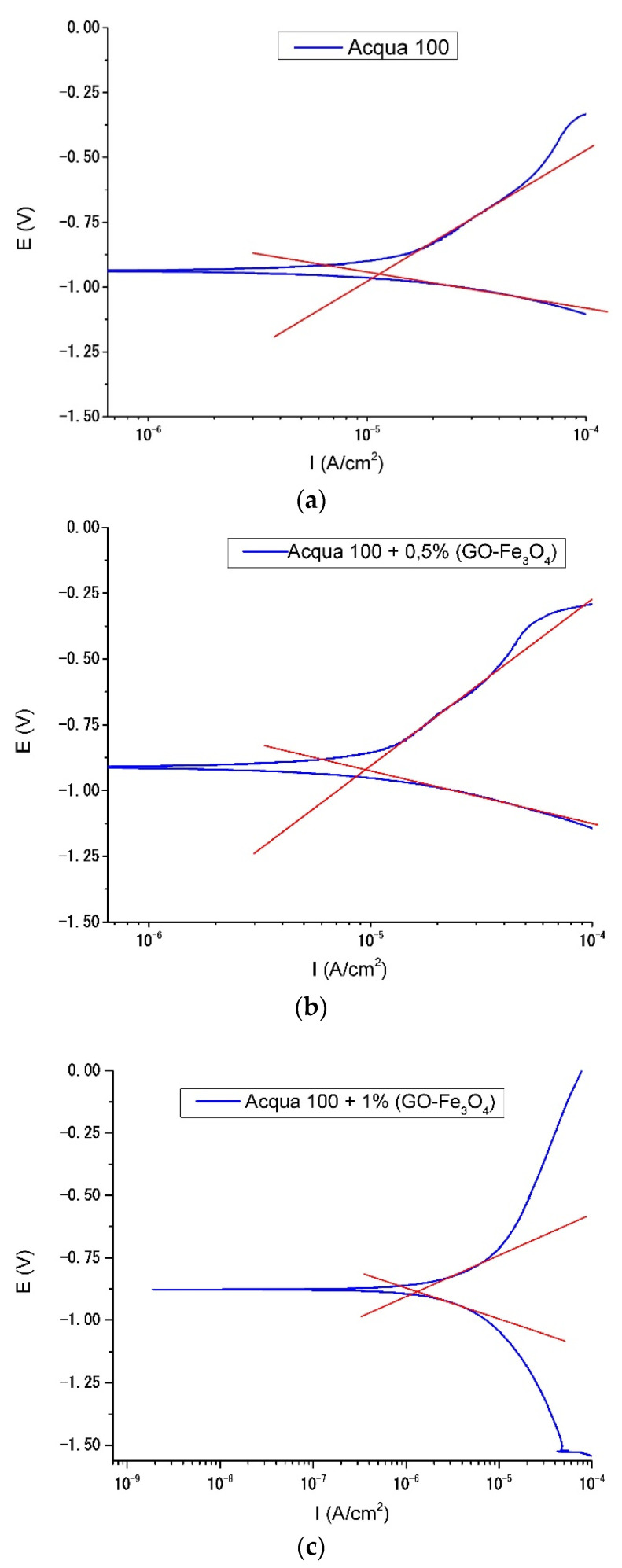
(**a**). Polarization curve for (Acqua 100) with corresponding settings. (**b**). Polarization curve for (Acqua 100 + 0.5% NPs) with corresponding settings. (**c**). Polarization curve for (Acqua 100 + 1% NPs) with corresponding settings.

The results of the potentiodynamic polarization curve tests are shown in Figure 16a–c. The blue lines correspond to the potentiodynamic curves for the samples with Acqua 100, Acqua 100 + 0.5%, and Acqua 100 + 1%,. Meanwhile, the red lines correspond to the intersection of the linear part of the anodic reaction curve with the cathodic reaction curve for each figure.

After processing and analyzing the data, the corrosion rate and mass loss are calculated. The results are provided in Table 6, where CR is the corrosion rate and is given in millimeters per year (mm/y), and MR is the mass loss rate and is expressed in grams per square meter day (g/m^2^d).

According to the results obtained with the potentiodynamic test, it can be concluded that the nanoparticle incorporation into the Acqua 100 coating improves the corrosion rate and mass loss parameters. It was found at 1 wt. % of NPs addition improvements of 85% for the corrosion rate and 89% for the mass loss compared with the Acqua 100 coating without NPs. Additionally, it should be noted that samples with 0.5 wt. % of addition of NPs obtained better improvement than the sample coating without NPs.

Table 7 shows the average values of the results obtained with the abrasion test. The samples with 1 wt. % of NPs show a considerable improvement of 16% higher resistance to abrasion than the Acqua 100 samples (without NPs).

## 4. Conclusions

Magnetite particles with superparamagnetic characteristics were successfully synthesized at a nanometer scale. Particle sizes between 3 and 12 nm at a temperature range between 25 °C and 95 °C were obtained. Magnetite nanoparticles have rhombohedral and orthorhombic crystallinity (according to TEM and XRD analysis). Magnetite nanoparticles synthesized at 95 °C (with an average size distribution of 3 nm) showed a quasi-spherical shape and a rhombohedral crystallinity. Moreover, the particle sizes could be controlled by adjusting the reaction temperature. As was observed, the magnetite nanoparticle sizes increase with increasing temperature.

The biosynthesis was carried out in a single step by chemical co-precipitation and green chemistry method using extracts of green lemon peel.

Thanks to the co-precipitation green method, the Fe_3_O_4_ nanoparticle processing was an achievement. Moreover, a certain crystallinity was obtained. It allowed the inhibition and the control of the growth, as well as the stabilization of magnetite nanoparticles during the reaction process [54].

The extracts from lemon peel can act as reducing and stabilizing agents in nanoparticle synthesis, which might be due to the presence of phytochemicals. Phytochemicals are compounds produced from the lemon plant itself. They contain phytochemicals such as limonene, flavonoid, xanthophylls, carotenoids, anthocyanins, and polyphenolic and ascorbic acids that participate in nanoparticle synthesis [33,66]. The D Limonene compound became a new and original surfactant agent [35]. It controls the nucleation and growth process of the co-precipitation of magnetite nanoparticles. Thus, smaller particles were successfully obtained in the present work.

On the other hand, to improve the green synthesis of nanoparticles and make it more eco-friendly, we suggest changing the NaOH base for sodium carbonate in combination with green chemistry in the future [67].

A corrosion rate of 0.02 mm/yr was obtained for the samples coated with Acqua 100 + 1 wt. % of nanoparticles. This value is 85% lower than that of samples coated with Acqua 100. The mass loss was 0.33 g/m^2^d, i.e., 89% lower than that of samples coated with Acqua 100. Concerning the tribology tests, the samples coated with Acqua 100 + 1 wt. % of nanoparticles presented the highest values of resistance factor. This is 16% higher than the value of the samples coated with Acqua 100.

In this way, we can claim that the coating developed with the nanoparticles synthesized by the green method has good anticorrosive properties. This characteristic makes it a promising candidate for the preparation of anticorrosive coatings.

This research line will continue with anticorrosive coatings studies and expand the conditions used in the synthesis and preparation of new composites. Additionally, the utilization of other techniques for evaluating and characterizing the coating is recommended. Other studies on corrosive techniques can provide us with more information about coating properties. The development of anticorrosive coatings is attractive for industrial and structural-grade steel applications.

## Figures and Tables

**Table 1:** Synthesis of magnetic nanoparticles at different temperatures.

Characteristic Experiment	Ratio Fe^3+^:Fe^2+^	Temperature
M1	2:1	25 °C
M2	2:1	55 °C
M3	2:1	85 °C
M4	2:1	95 °C

**Table 2:** Materials used in the preparation of the composite.

NPs (M4)	GO	DMF	H_2_O	Ethyl Alcohol	Acqua 100
0.2 g	1 g	400 mL	400 mL	600 mL	300 g

**Table 3:** Some specific characteristics of samples of iron oxide: phase, color, observed intensity indexes, comparison with JCPDS card numbers, and the average size from the Sherrer equation.

Sample	Iron Oxide Phase	Color	Observed Intensity Index	Comparison with JCPDS Card No.	Average Size (Scherrer)
M1	Rhombohedral-Fe_3_O_4_	Dark brown	(110) (220), (222) (311), (400) (422), (104) (511),	00-019-0629	3 nm
M2	Rhombohedral-Fe_3_O_4_	Dark brown	(104) (110), (021) (113), (033) (125), (208) (220)	01-071-6766	7 nm
M3	Orthorhombic-Fe_3_O_4_	Dark brown	(020) (114), (212) (122), (314) (028), (322) (110), (228) (040),	01-076-0955	10 nm
M4	Orthorhombic-Fe_3_O_4_	Dark brown	(110) (004), (106) (122), (026) (222), (040) (228)	01-076-0957	12 nm

**Table 4:** Parameter of Fe_3_O_4_ nanoparticles obtained from XRD patterns of M1 sample and JCPDS standard Magnetite No. 00-19-0629 [51].

2ϴ	Miller Index hkl	d_hkl_ (Å) JCPDS 00-19-0629	d_hkl_ (Å) Experimental	Percentage Relative Error $e=\frac{\left|Exp-Theor\right|}{Theor}\times 100$
30.5	(220)	2.97	2.92	2.02%
35.6	(311)	2.53	2.51	0.79%
43.32	(400)	2.10	2.08	0.95%
53.96	(422)	1.71	1.69	1.69%
57.54	(511)	1.62	1.59	1.85%
63.14	(440)	1.49	1.47	1.34%

**Table 5:** Average values of corrosion potential.

Acqua 100	Acqua 100 + 0.5% de NPs	Acqua 100 + 1% de NPs
−0.6578 V	−0.6483 V	−0.6158 V

**Table 6:** Comparative results potentiodynamic test.

	Acqua 100	Acqua 100 + 0.5 wt.%	Acqua 100 + 1 wt.%
**CR (mm/y)**	0.14	0.06	0.02
**MR (g/m^2^d)**	3.07	1.32	0.33

**Table 7:** Tribology Test Values.

Samples	Thickness (μm)	Volume (L)	Resistance Factor
Acqua 100	67.80	156	2.3
Acqua 100 + 0.5%	69.34	178	2.56
Acqua 100 + 1%	83.82	222	2.67

## Data Availability

The data presented in this study are available on request from the corresponding author.
